# Tau‐induced astrocyte senescence and dysfunction in AD tauopathy

**DOI:** 10.1002/alz.093341

**Published:** 2025-01-03

**Authors:** Haneen Makhlouf

**Affiliations:** ^1^ University of Oklahoma Health Sciences Center, Oklahoma City, OK USA

## Abstract

**Background:**

Cellular senescence is defined as cell cycle arrest and the acquisition of a proinflammatory ‘senescence‐associated secretory phenotype’ (SASP). In Alzheimer’s disease (AD), tau protein in neurons undergoes hyperphosphorylation and misfolding, resulting in the formation of pathogenic soluble aggregates known as tau oligomers. Tau oligomers are released from neurons during neuronal activation and are transmitted to postsynaptic cells in a prion‐like fashion. In addition to neurons, tau is expressed in various brain cell types, including astrocytes. In the present study, we tested the central hypothesis that soluble pathogenic tau can be transmitted to astrocytes and that tau transmission to astrocytes induces cytoskeleton destabilization and cellular dysfunction, contributing to the progression of AD.

**Methods:**

to test the molecular consequences of tau transmission, we exposed primary human astrocytes with purified tau oligomers and we measured markers of senescence (cell and nuclear size, cell cycle arrest, and SASP activation) using advanced biochemical tools, immunocytochemistry, and live‐cell microscopy. We also measured cell cycle arrest and SASP activation in the whole brain and astrocyte fractions isolated from hTau mice, a mouse model of AD tauopathy. In addition, we co‐cultured human astrocytes undergoing tau‐induced senescence with primary mouse or non‐human primate neurons to determine the impact of senescent astrocytes on neurons (using immunocytochemistry and 3D image analyses to measure aspects of dendrite and spine abundance and morphology).

**Results:**

Our findings reveal that pathogenic soluble tau is readily transmitted to primary human astrocytes *in vitro* and *in vivo* and that pathogenic tau transmission triggers phosphorylation of endogenous astrocyte tau, leading to the destabilization of the microtubule cytoskeleton. Further, we found that transmission of pathogenic tau to astrocytes potently upregulated multiple markers of cellular senescence, demonstrating for the first time pathogenic tau‐induced astrocyte senescence. Moreover, density of dendritic spines and dendritic area was pronouncedly decreased in neurons exposed to astrocytes undergoing tau‐induced senescence.

**Conclusion:**

Our study showed that pathogenic tau is transmitted to cultured human astrocytes and that transmission of pathogenic tau to astrocytes triggers astrocyte senescence in a mouse model of AD tauopathy. Our data suggest that senescent astrocytes may be critical mediators of neuronal dysfunction in AD tauopathy.

